# Circadian rhythms in cardiovascular disease

**DOI:** 10.1093/eurheartj/ehaf367

**Published:** 2025-07-15

**Authors:** Ilse R Kelters, Yvonne Koop, Martin E Young, Andreas Daiber, Linda W van Laake

**Affiliations:** Department of Cardiology and Experimental Cardiology Laboratory, Division of Heart & Lungs, University Medical Centre Utrecht, Heidelberglaan 11, 3584 CX Utrecht, The Netherlands; Cardiovascular Epidemiology, Julius Center for Health Sciences and Primary Care, Utrecht University Medical Centre, Utrecht University, Utrecht, The Netherlands; Department of Oncology-Pathology, Karolinska Institutet, Stockholm, Sweden; Division of Cardiovascular Disease, Department of Medicine, University of Alabama at Birmingham, Birmingham, AL, USA; University Medical Center Mainz, Department of Cardiology 1, Johannes Gutenberg University, Langenbeckstr. 1 Mainz 55131, Germany; Center for Cardiology, Molecular Cardiology, German Center for Cardiovascular Research (DZHK), Partner Site Rhine-Main, Mainz, Germany; Department of Cardiology and Experimental Cardiology Laboratory, Division of Heart & Lungs, University Medical Centre Utrecht, Heidelberglaan 11, 3584 CX Utrecht, The Netherlands

**Keywords:** Circadian Rhythms, Cardiovascular Disease, Chronotherapy, Chronomodulation, Heart Failure, ischaemic Heart Disease, Myocardial Infarction, Epidemiology, Incidence

## Abstract

Circadian rhythms, controlled by the suprachiasmatic nucleus and peripheral clocks, regulate 24-h cycles in biological processes such as the cardiovascular system. Circadian rhythms influence autonomic balance, with parasympathetic dominance during sleep supporting cardiac recovery and sympathetic activation during the day supporting circulatory demand. Congruent with systemic and cellular circadian rhythmicity, 24-h patterns arise in the pathophysiology of cardiovascular diseases, including ischaemic heart disease, heart failure, and arrhythmias.

Daily variations influence the timing and outcome of myocardial infarction, with studies reporting patterns in infarct size depending on the time of onset. Similar daily patterns are observed in cardio- and cerebrovascular complications. In heart failure, circadian rhythms are dampened but remain intact, suggesting the potential for incorporating timing in diagnostics and therapies. Sudden cardiac death follows a distinct pattern, with a higher incidence in the morning. Atrial fibrillation onset, on the other hand, occurs more frequently at night.

Risk factors and modifiers, such as physiological, psychological, lifestyle, and environmental factors and comorbidities interact with circadian rhythms, thereby impacting cellular pathomechanisms and development of cardiovascular health and disease. Chronotherapy, which aligns treatments with circadian rhythms, has demonstrated potential for improving the efficacy of cardiovascular therapies. This review examines the influence of circadian rhythms on cardiovascular health in the context of specific cardiac diseases and risk factors, and it highlights the therapeutic opportunities informed by circadian patterns.

## Graphical Abstract

**Graphical Abstract:**
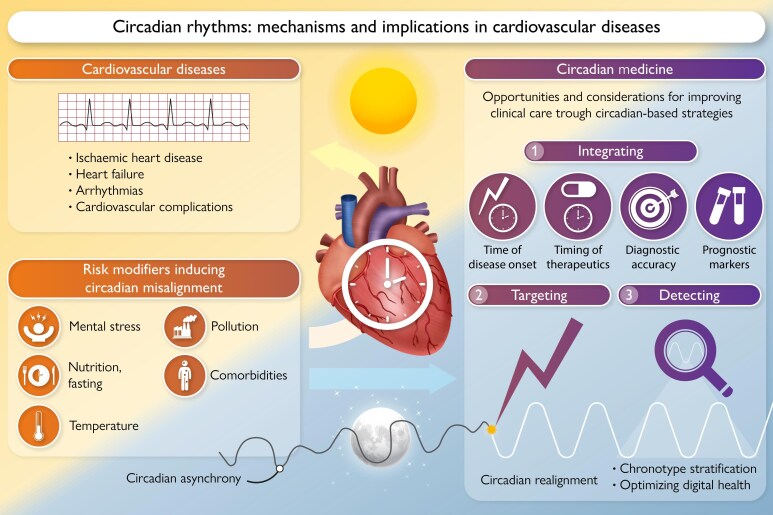
Circadian rhythms have an impact on cardiac disease, several evironmental and behavioural factors can induce circadian misalignment. Several opportunities can be considered to integrate circadian-based strategies, and to target and detect circadian rhythm variation.

## Introduction

Circadian rhythms are endogenously driven 24 h cycles in biological processes that enable organisms to anticipate and adapt to the natural day-night light cycle. Circadian rhythms (Box 1) are governed by a hierarchical system of biological clocks, with the suprachiasmatic nucleus (SCN) in the hypothalamus serving as the central pacemaker. The SCN synchronises peripheral clocks present in virtually all nucleated cells, including those in cardiovascular tissues, through neural, humoral, and behavioural signals such as sleep, food intake, and physical activity.^1,2^

Box 1 **Overview of terminology**

**Table:** 

Term	Definition
Circadian rhythm	An approximately 24-h cycle in biological processes, regulated by internal clocks and influenced by external factors such as light and temperature.
Circadian clock	The internal biological mechanism that regulates circadian rhythms, primarily controlled by the suprachiasmatic nucleus (SCN) in the brain, which synchronizes physiological and behavioural processes.
Peripheral clock	Clocks present in tissues and organs outside the SCN, which help regulate local physiological functions and can be influenced by external cues like food intake and physical activity.
Diurnal rhythm	Patterns in behaviour or physiology that occur during the daytime and are influenced by external environmental factors, such as light and temperature, rather than solely by internal biological clocks.
Daily patterns	Recurring physiological or behavioural changes that follow a roughly 24-h cycle, such as sleep-wake cycles, hormone release, and body temperature fluctuations.
Chronotype	An individual's natural preference for sleep and activity timing, ranging from morning types to evening types, influenced by genetic and environmental factors.

**Figure 1:**
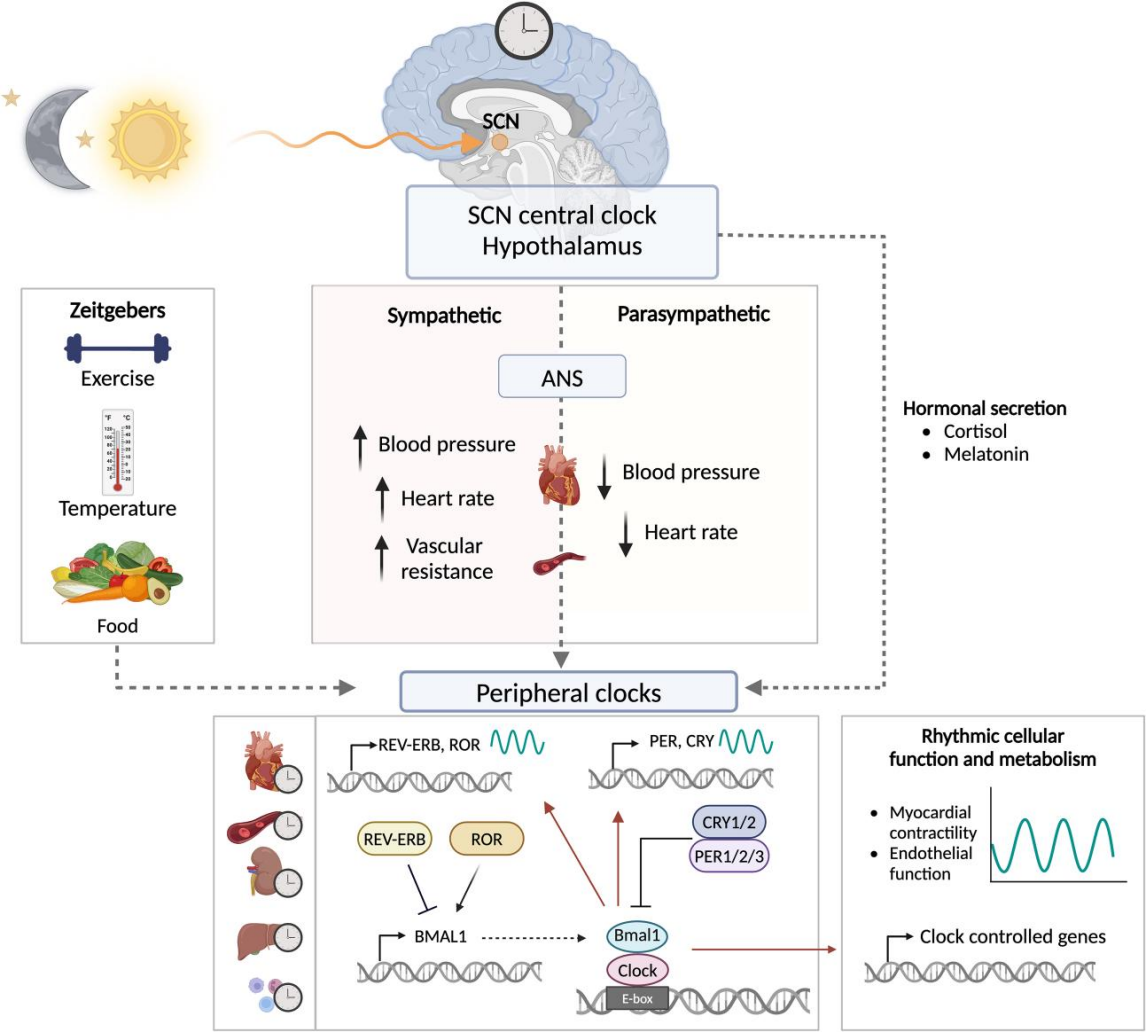
Circadian regulation of molecular clock gene signalling and physiological regulation. Circadian regulation of molecular clock gene signalling and physiological processes. This overview illustrates the circadian control of molecular clock genes, clock-controlled genes, and physiological functions, including the autonomic nervous system's influence on heart rate variability, heart rate, and blood pressure. ANS, autonomic nervous system; SCN, suprachiasmatic nucleus

At the molecular level, circadian rhythms are generated by a molecular clock, which consists of multiple core clock proteins including circadian locomotor output cycles kaput (CLOCK), brain and muscle ARNT-Like 1, cryptochrome 1/2 (CRY1/2), period 1/2/3 (PER1/2/3), retinoic acid receptor orphan receptor α/β/γ (RORα/β/γ), and nuclear receptor 1D1/2 (REV-ERBα/β)(*Figure 1*).^3^ The core clock machinery regulates rhythmic fluctuations in clock-controlled genes expression via transcriptional–translational feedback loops.^3^ The oscillation in expression of those genes induces a 24 h cycle in processes including myocardial contractility, cell metabolism, and endothelial function.^1,2^ Both the sympathetic and parasympathetic nerve systems directly and indirectly affect circadian clock function,^4,5^ linking sleep to circadian clock function as a mediating factor.

Sleep plays an important role in maintaining 24 h rhythms and cardiovascular homeostasis. During sleep, the parasympathetic system dominance lowers heart rate (HR) and blood pressure (BP), while reduction in sympathetic tone decreases vascular resistance, collectively supporting cardiac recovery. The sympathetic system becomes more active during the day, increasing HR and BP. These fluctuations are linked with daily patterns in disease onset.^6^

**Figure 2:**
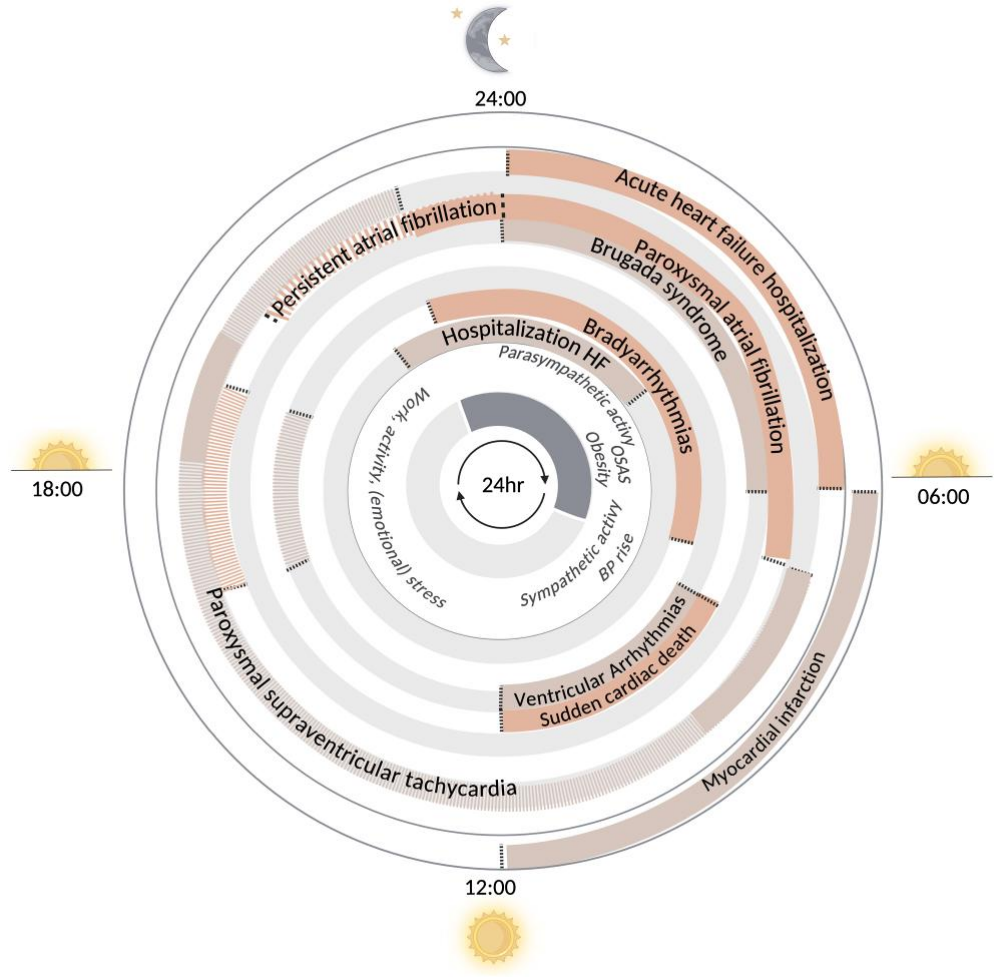
Incidence peaks of cardiovascular diseases during a 24-h period. A circular visualization summarizing the incidence peaks of various cardiovascular diseases. Timepoints corresponding to incidence peaks for several cardiovascular diseases are illustrated, with supporting literature provided in Supplementary data online, *Tables S2*–*S4*. Dashed lines represent second-highest peak of incidence. BP, blood pressure; HF, heart failure; hr, hour; OSAS, obstructive sleep apnoea

The early morning transition from sleep to awake increases sympathetic activity, coinciding with an increased risk of events, such as myocardial infarction (MI) (*Figure 2*).^7^ Circadian rhythm disruptions—such as shift work, sleep deprivation, or irregular meal timing—impair molecular clock pathways, increasing the risk of cardiovascular diseases (CVD) such as hypertension, atherosclerosis, and arrhythmias.^7,8^

Heart rate variability (HRV)—reflecting autonomic nervous system regulation—follows a daily pattern influenced by the SCN through its control of the parasympathetic and sympathetic branches. Additionally, peripheral circadian clocks in cardiovascular tissues, such as myocardium and blood vessels, contribute to variations in cardiovascular function. The daily variations in HRV reflect the combined actions of the SCN and peripheral clocks,^1^ with sympathetic regulation further supporting circadian clocks within the cardiovascular system.

This interplay extends to therapeutic strategies. Chronotherapy, the timing of treatments to align with the body's biological rhythms, has shown promise in improving the effectiveness of medications such as antihypertensives and anticoagulation.^9–11^ Understanding the mechanisms by which circadian rhythms regulate cardiovascular (patho)physiology offers an opportunity to refine prevention and treatment strategies.

This review summarizes the interplay between circadian biology and cardiovascular function in the context of cardiac diseases, highlighting clinical implications, diurnal variations in disease onset, and the modulation of the circadian interactome by internal and external risk factors. We discuss how disrupted rhythms increase cardiovascular risk and their potential clinical and research recommendations as outlined in *Table 1*.

**Table 1:** Recommendations for clinical care, studies and drug development

**Clinical care**
Registration of time in intervention, drug administration, physiological and laboratory measurements Timing of diagnostics considering optimal predictive value Optimalization of clinical care organization considering common time of onset of disease 24 h ambulatory BP and HR measurements for cardiovascular risk Minimalize disruption of daily rhythms and sleep in clinical patients Minimalize health hazards of clinical staff due to shift work—provide preventive programme
**Future** Time-of-day-adjusted clinical reference ranges Biomarkers for functioning of the circadian system Optimizing clinical procedures considering time-of-day
**Clinical studies**
Categorize sex, age, and ethnicity Consider the effect of cardiovascular risk factors on the circadian system (obesity, diabetes mellitus, etc.) Consider confounding effects of light exposure, sleeping conditions, noise, exercise, diet Stratify or standardize time of intervention Stratify or standardize time of diagnosis Stratify or standardize time of sample collection Use more than two timepoints of intervention, medication application and/or (intermediate outcome) measurement
**Drug development**
Standardize circadian timing in *in vitro* and *in vivo* studies Study multiple time points Considering the influence of circadian rhythms on pharmacokinetics and dynamics Consider choice of animal model (diurnal, nocturnal) and translate to human rhythm if applicable Standardize relevant housing rhythm (e.g. light:dark schedule, temperature) Choose appropriate sex and age Align time of feeding Consider time of sample collection: standardize Consider time of intervention (e.g. drug delivery, surgery): standardize Use more than two timepoints for intervention and/or sampling 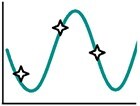

## Methods

The methods for this narrative review are described in the supplemental material (see Supplementary data online, *Methods* and *Table S1*).

## Results

### Circadian influences on ischaemic heart disease

#### Daily patterns and myocardial infarction risk

Myocardial infarctions (MIs) occur more frequently in the morning (*Figure 2*), a pattern reflecting 24 h rhythms in cardiovascular functions, such as BP and coagulation. These rhythms influence platelet aggregation, which increases in the morning, alongside higher BP, leading to a higher risk of thromboembolic events. Studies found that the incidence of MI is three times higher in the morning compared to late evening.^12–14^ Moreover, ST segment elevation myocardial infarction (STEMI) was ∼10% more common in the morning than in the afternoon or night.^15^ Myocardial infarction with non-obstructive coronary arteries follows a similar 24 h pattern to MI with obstructive coronary arteries, with higher incidence in the morning.^16^ The time-of-day variations in MI incidence are outlined in Supplementary data online, *Table S2*. Daylight saving time transitions have been linked to a rise in MI incidence, particularly at the onset of daylight saving time in spring especially during the week after transition, with an increase in non-STEMI cases.^17^ The week after the autumn shift shows no significant increase or decrease in MI incidence.^17,18^ Sleep quality and duration are affected and MI incidence is increased for 3 weeks after the time reset, with a more pronounced difference in women,^18^ a subsequent meta-analysis supported these findings.^19^ However, sleep may not be the only determinant for the observed higher cardiovascular risk, as the general environmental condition, gender and individual preference in circadian rhythms (chronotype) may play a role. Evening chronotypes, for example, are more prone to sleep-related issues, unhealthy lifestyle habits, and metabolic disturbances, all of which are linked to cardiovascular risk.^20^ Additionally, women with an evening chronotype appear to be more susceptible to sleep disruption and related health consequences, which could further influence cardiovascular outcomes.^20^ These findings highlight the importance of considering time-of-day and external triggers in the management of ischaemic heart disease (IHD).

#### Infarct size and prognosis

Preclinical studies in mice have shown that ischaemia during the sleep-to-wake transition results in larger infarcts and more severe outcomes.^21^ Clinical studies were initiated to validate these findings using methods like cardiovascular magnetic resonance, echocardiography, and cardiac biomarkers such as creatine kinase and troponin-I. Biomarkers and imaging also reflect circadian differences in myocardial injury, with larger infarcts observed in the morning.^22^ A study analysing four randomized trials found a circadian variation in myocardial infarct size, with the largest infarcts between midnight and 06:00, suggesting that symptom onset timing impacts infarct size and potentially future treatments.^23^ Another study using tissue Doppler echocardiography showed the largest infarcts and worst left ventricular function after an MI between 06:00 and noon,^24^ a result that was confirmed by an analysis of 1031 STEMI patients revealing that both infarct size and left ventricular function were time-of-day-dependent, with the most severe effects observed during early morning hours.^25^ The differences between these studies may reflect variations in study design, population characteristics, or definitions of time windows, but collectively current evidence suggests that infarct size is highest between midnight and noon. Several studies have explored the impact of symptom onset timing in STEMI patients on long-term prognosis. One found that late evening or early morning symptom onset increased the risk of rehospitalization for heart failure (HF) by 30%–50% after 1 year, particularly if ischaemic duration exceeded 120 min.^26^ Another study found that night-onset STEMI was associated with higher mortality.^27^ Additionally, patients with early morning symptom onset had larger infarct sizes and higher 30-day mortality rates.^28^

A randomized study on aortic valve replacement surgery, where a brief ischaemic period is induced, revealed that afternoon surgeries entail less myocardial injury, possibly due to circadian patterns influencing myocardial tolerance to ischaemia.^29^ However, not all studies confirmed a daytime dependence in elective cardiac surgery for either aortic valve replacement or coronary artery bypass grafting and clinical outcome.^30–32^ Given the substantial knowledge on the importance of the time-of-day for cardioprotective interventions, circadian regulation of central processes should be considered in future research and clinical practice.^33^

#### Diagnostic tools

Patients with nocturnal ST segment changes, detected through dynamic electrocardiogram (ECG) monitoring in individuals undergoing coronary angiography, showed a significantly higher chance of having IHD (93%) compared to those without such changes (22%). Particularly, nightly peak-shaped ST segment changes were strongly associated with IHD.^34^ These results might provide an opportunity for using a non-invasive 24-h diagnostic tool for IHD.

For MI diagnosis cardiac troponin-I shows no significant diurnal fluctuation between morning (23:00 to 14:00) and evening (14:00 to 23:00) presentations of MI, indicating that its diagnostic accuracy remains consistent regardless of the time of presentation.^35^ In contrast, troponin-T exhibits a distinct pattern, with concentrations gradually declining during daytime and rising overnight, peaking in the early morning hours.^36,37^ While this pattern does not impact the diagnostic accuracy for MI, it could influence the interpretation of absolute changes in troponin levels within the first hour of presentation suggesting that interpretation in non-MI settings should be informed by time of sampling.^36^

#### Clinical evidence

Unlike preclinical studies, clinical evidence on circadian biology in MI remains insufficient, as studies have been observational, focussing on time-dependent patterns (e.g. time-of-day variations in MI incidence) rather than endogenous circadian mechanisms.^38^

### Circadian influences in heart failure

#### Disruption of circadian biology in heart failure

HF is a global health issue, with circadian disruption contributing to its progression. Circadian clocks regulate factors relevant in the pathophysiology of HF including HR, BP, myocardial contractility, and inflammation. Preclinical studies suggest that disruptions in these clocks contribute to declining cardiac function, impaired neurohormonal regulation, and disturbed sleep-wake cycles.^6,39^ Research highlights that time-of-day fluctuations in cardiac contractility and electrical activity are less pronounced in HF patients, driven by altered neurohormonal rhythms and reduced ability of the heart to efficiently perform its functions, such as maintaining blood flow and oxygen delivery, particularly during the night when myocardial growth and renewal processes are more active.^40^ Deviations of normal 24-h rhythmicity, indicated by elevated nocturnal HR and BP, increase the risk of arrhythmias. Sleep deprivation, which is common in HF, exacerbates HF via increased sympathetic activity and inflammation. Insomnia, obstructive sleep apnoea syndrome (OSAS), shift work, and non-dipping hypertension worsen HF, by triggering neurohormonal pathways and abnormal BP fluctuations.^40–42^ Circadian clocks also influence cardiovascular growth and remodelling, particularly during sleep when HR and BP are normally lowest. Disruptions in these mechanisms likely elevate cardiovascular risk and may worsen HF outcomes.^43^

#### Diagnostic opportunities

Diagnosing disrupted 24 h patterns during HF involves detecting altered sleep patterns, electrophysiology, and neurohormonal imbalances. Recent advancements, including wearable devices, improve continuous monitoring by measuring HRV and sleep patterns in real-time. Innovative diagnostic methods further highlight the impact of time-of-day in HF diagnosis. A deep learning model integrating time, frequency, non-linear and fragmentation HRV parameters from 24-hr ECG recordings achieved high accuracy (92.6%), sensitivity (90.7%), and specificity (95.2%), particularly in diagnosing HF with preserved ejection fraction (HFpEF).^44^ A colour-coded polar representation combining HRV and patient profiles showed 93% accuracy, complementing echocardiography by offering insights into 24 h patterns.^44^ Time-specific ECG-based regression models used daily fluctuations to improve left ventricular ejection fraction estimation.^45^ Nighttime HR patterns linked to increased HF risk in the JAMP study emphasize the need for nighttime haemodynamic assessments.^46^ Furthermore, biomarker research revealed diurnal BNP and NT-proBNP variations improving acute HF diagnosis, while ambulatory BP monitoring identified non-dippers, enhancing prognostic evaluations.^47,48^ Interestingly, ambulatory invasive monitoring using CardioMEMS revealed diurnal variation in pulmonary artery pressures with the lowest and most stable values in the morning,^49^ emphasizing standardization of time of measurement.

#### Prognostic implications

Circadian rhythm disruption may have prognostic implications for HF patients. The central circadian clock, reflected by melatonin and cortisol levels, is dampened but intact in HF patients, emphasizing the potential opportunity of timing in clinical interventions to improve outcomes.^37^ Persistent circadian misalignment, referring to either a blunted day-night variation or a shifted rhythm in physiological processes such as BP and HR regulation, significantly worsens prognosis.^39^ Not being able to maintain normal circadian rhythms, as reflected by disrupted sleep, meal timing, and BP patterns, not only increases HF risk but also exacerbates the condition once it develops.^41,43^

Wrist-based BP monitoring provides accurate nighttime BP assessment with less sleep disruption compared to upper-arm measurement, potentially improving hypertension management in these patients.^50^ Furthermore, BP variability and non-dipping patterns are strong predictors of cardiovascular events in young hypertensive individuals,^51^ while nocturnal thoracic volume overload and abnormal circadian BP patterns–particularly the riser and non-dipper–are critical predictors of poorer outcomes in acute HF.^52,53^

#### Therapeutic interventions

Therapeutic strategies, most recently sodium-glucose cotransporter 2 inhibitors have been reported to improve cardiac autonomic function, reducing daytime and nighttime HRs while enhancing parasympathetic modulation and exercise capacity, which may normalize circadian rhythm.^54^ Beta-blockers enhance HRV by suppressing sympathetic activity, leading to an (indirect) increase in parasympathetic activity, particularly during high-risk morning hours, potentially supporting cardiac autonomic regulation in HFpEF patients.^55^ Beta-blockers may suppress melatonin production, and while melatonin supplementation might have benefits for sleep quality in patients on beta-blockers there is limited evidence on its clinical benefits in this population.^56^ Angiotensin-converting enzyme (ACE) inhibitors restore circadian BP variability, improving clinical status in congestive HF.^57^ Moreover, digoxin treatment reduces sympathetic activity during sleep, modulating BP and parasympathetic function.^58^ Advanced technologies, such as left ventricular assist devices and high-frequency pump monitoring, also highlight the importance of understanding 24 h rhythms in managing HF: Diurnal rhythmicity was demonstrated in pump parameters even though the technical settings are fixed, thus reflecting circadian physiology in patient-pump interaction.^59^

Heart transplantation involves circadian interactions between donor and recipient, affecting graft function, immune response, and ischaemia-reperfusion injury.^60–62^ Moreover, cardiac sympathetic reinnervation is associated with improved survival rates in heart transplant patients, highlighting the beneficial influence of the autonomic nervous system.^63^ The impact of donor heart procurement time or re-implantation timing on transplant viability, in relation to patient prognostics, is difficult to investigate due to multiple confounding factors (wakefulness/focus of the surgeon, logistics, etc.). One study found that donor hearts procured in the early morning between 04:00 and 11:00 were associated with better long-term survival rates, while nighttime surgeries were linked to worse outcomes.^60^

### Circadian rhythms in electrophysiology and arrhythmias

Daily rhythms are present in cardiac electrophysiology, including sinus node function, myocardial refractoriness, QT interval, QRS duration, and PR interval. These rhythms are regulated by the autonomic nervous system via the SCN, and the peripheral cardiac cell clock governing ion channel transcription and function.^64,65^

During the night the autonomic tone shifts towards parasympathetic (vagal) dominance, facilitating relatively benign bradyarrhythmias such as sinus bradycardia and atrioventricular block with prolongation of PR, QT, and QRS intervals.^66^ Conversely, the early morning hours are characterized by a surge in sympathetic activity, involving increase in circulating levels of catecholamines. This sympathetic activation, along with concurrent increases in HR, BP, and myocardial oxygen demand, are thought to increase the risk of ventricular arrhythmias (VA) by triggering abnormalities in intracellular calcium handling and action potential repolarization.^67^ Another actively studied mechanism underlying the susceptibility of VA in the morning hours is the circadian clock dependent modulation of electrical activity driven by functional changes of cardiac ion channels and extrinsic autonomic receptor mediated mechanisms (reviewed by Bernardi *et al.*^68^). Multiple ion channels subunits exhibit distinct 24-h mRNA and/or protein level oscillation, which are abolished upon disruption of the cardiomyocyte circadian clock.^68^ Moreover, neurohumoral factors regulate cardiac electrophysiology by modulating ion channel expression. During the morning corticosterone peak, increased binding to the circadian clock-regulated glucocorticoid receptor enhances the expression of ion channel subunits Scn5a and Kcnh2 and corresponding ionic currents I_na_ and I_kr_, along with the gap junction protein Connexin 43.^69^ These changes alter electrophysiological properties in response to hormonal signals potentially increasing arrhythmia susceptibility in during the active phase.^69^

In humans, ion channel expression also follows day-night rhythms.^70^ However, it is still unknown how combination of these ion channel subunits rhythms impacts the ventricular action potential.

Daily patterns in cardiac electrophysiology are reflected by epidemiological studies, with a well-documented peak of VA incidence in the morning (07:00–12:00), mirroring sudden cardiac death patterns (*Figure 2*; Supplementary data online, *Table S3*). Variations in study populations and advances in pharmacological management have highlighted factors that attenuate this pattern, including comorbidities (chronic kidney disease, diabetes mellitus^71,72^), beta-blockers blunting autonomic surges, and advanced HF therapies.^73–75^ Notably, ventricular tachyarrhythmias in patients with OSAS, Brugada and early repolarization syndrome often occur nocturnally.^76–81^ This is linked to elevated nocturnal sympathetic- and reductions parasympathetic tone in OSAS patients.^82^

For atrial fibrillation (AF), the interplay between autonomic inputs and tim- of-day is more complex (see Supplementary data online, *Table S4*). A subset of patients exhibit a predominant or even exclusive nocturnal onset of AF, potentially driven by vagal tone.^83^ This parasympathetic activity shortens the atrial effective refractory period by activation of the acetylcholine-activated outward potassium current, and promotes electrical remodelling, thereby increasing the propensity for re-entry circuits.^84^ In contrast, AF episodes during the day are often triggered by adrenergic surges (physical or emotional stress), promoting arrhythmogenesis by boosting calcium dependent cardiac function via L-type calcium channels leading to early afterdepolarizations.^85,86^ However, direct sympathetic and parasympathetic nerve activity recordings in canine models have demonstrated that AF initiation is often preceded by concurrent increases in both autonomic outputs.^87^ This finding suggests that autonomic co-activation may play a pivotal role, underscoring the complex and multifactorial nature of AF initiation. Additionally, sleep-related disorders, such as OSAS, exacerbate nocturnal autonomic dysregulation by introducing intermittent hypoxia and sympathetic bursts, further increasing AF susceptibility during sleep.^86,88^

A recent literature review reported a periodicity analysis to support the hypothesis of nocturnal onset subtype of AF in more detail.^83^ In total 2080 patients with 11 886 onsets of AF were included from 14 studies and a cosine fitting curve (*R*^2^ .77; *P* = .03) with a 24 h period was plotted. Significantly more onset of AF was detected during the night (22:00–07:00) compared to the daytime (07:00–22:00). Additionally, the effect of specific sleep stages (e.g. shorter slow-wave sleep), sleep-disordered breathing, and the effect of body positions on the onset of AF were described.^89–92^ In contrast, paroxysmal supraventricular arrhythmias are most frequent during the day, conceivably driven by cell-autonomous circadian rhythmicity in addition to sympathetic drive.^93,94^

#### Diagnostic, therapeutic and prognostic implications

Understanding daily variations in cardiac electrophysiology potentially has implications for diagnosis, treatment and prognosis. For instance, the QTc interval lengthens at night, and shortens with the morning autonomic surge, necessitating careful timing of ECG acquisition to avoid false-positive and false-negative diagnoses of long QT syndrome and drug induced QTc changes.^95^ Moreover, the extent of QTc interval prolongation after levofloxacin administration depends on dosing time, with the largest effect at 14:00 suggesting a time-adjusted strategy could improve safety in high-risk patients.^96^ When considering prognostic implications, time-of-day was an independent determinant of the corrected QT interval, with higher mortality risk in patients showing increased (peak-to-trough amplitude >15 ms) or diminished (peak-to-trough amplitude <5 ms) QT rhythmicity, as identified in a recent cohort study of 100 644 patients.^97^

### Circadian rhythms in other cardio- and cerebrovascular complications

The state of the central and peripheral clocks and the control they have over the physiology of the organism (HR, BP, peak cortisol in blood) are indicative of the chronobiological patterns seen in the incidence of cardiovascular events.^98^ A similar pattern exists for diverse cardiovascular complications. Rupture and dissection of aortic aneurysms are more likely to occur in the early morning,^99–101^ as well as ischaemic and haemorrhagic stroke.^102^ As these events represent completely different entities, similarity in chronobiological patterns is unexpected, but obviously all of them share the same circadian mechanisms.^103,104^

Another well-known cardiovascular complication is cancer therapy related cardiac damage, a leading cause of morbidity and mortality in cancer survivors. Anti-cancer therapies, such as anthracyclines, trastuzumab, taxanes, fluoropyrimidines, and tyrosine kinase inhibitors are well-documented for their cardiotoxic potential.^105^ While advancements in cardio-oncology have focused on mitigating these risks, the role of circadian rhythms in influencing cardiotoxicity and arrhythmogenesis is an emerging area of interest with substantial clinical implications.

The cardiomyocyte circadian clock governs numerous cellular processes, such as cardiac metabolism,^106^ DNA repair,^107^ cell fate,^108^ driving the time-dependent susceptibility to anthracycline-induced cardiomyocyte death observed *in vitro*.^109,110^ Preclinical studies further substantiate a circadian pattern in anthracycline-induced myocardial injury, with a protective effect observed when anthracyclines were administered around the transition from rest to activity phase,^111–113^ hinting towards a protective effect in the morning for humans. However, randomized controlled trials assessing the impact of chronomodulated chemotherapy administration on cardiotoxicity and therapeutic efficacy are still lacking.

### Modification of circadian rhythms and the interactome by risk factors and risk modifiers

**Figure 3:**
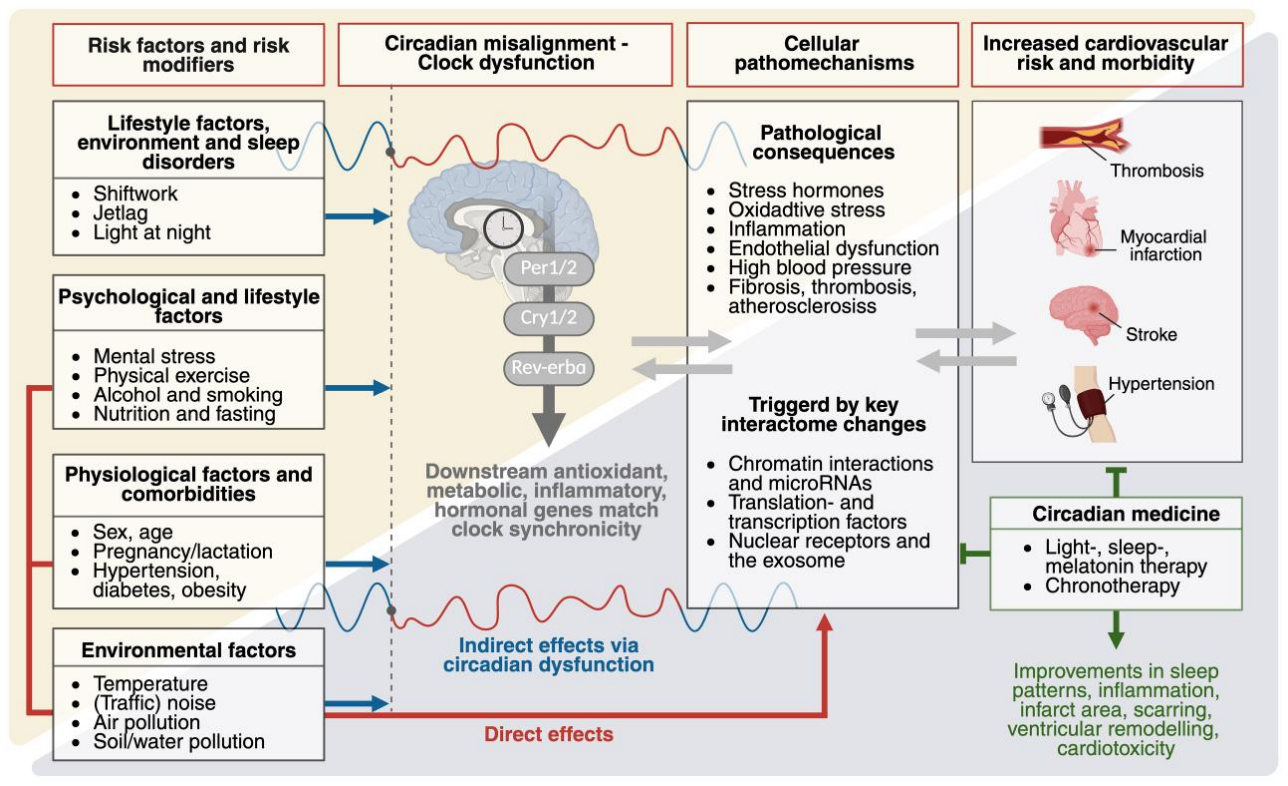
Overview of the circadian-cardiac interactome. An overview figure inspired by a directed acyclic graph, illustrating the key factors of the circadian-cardiac interactome. This visualization integrates the risk factors and risk modifiers influencing circadian rhythms and cardiac function. The red arrows indicate the direct influence of lifestyle, physiological, psychological and environmental factors on cardiovascular disease triggering key pathomechanisms by changes of key components of the interactome. Key pathomechanisms for cardiovascular diseases are higher levels in stress hormones, dysregulated expression of ROS producing and degrading enzymes leading to oxidative stress, activation of immune cells and increase in pro-inflammatory cytokines, exacerbated inflammation and progression of atherosclerosis, disturbed metabolism of glucose and fatty acids, impaired mitochondrial function (e.g. respiration, autophagy), dysregulated epigenetic pathways, enhanced thrombotic pathways and altered coagulation, phase shifts in blood pressure maximum and generally higher blood pressure levels, impaired vascular function, exacerbated arterial stiffness and promotion of fibrosis. The blue arrows indicate the influence of the risk factors and risk modifiers on circadian misalignment indirectly impacting cardiovascular risk. The black arrows indicate the influences between circadian misalignment, pathophysiology by interactome changes and cardiovascular disease. The latter can be beneficially influenced by chronotherapy approaches. CVD, cardiovascular disease; DAG, directed acyclic graph; SCN—suprachiasmatic nucleus;

Circadian interactomics describes the investigation of changes of circadian clock protein-protein interactions by various factors leading to alteration of the down-stream interactome, e.g. timekeeping of nuclear receptors and the exosome (mostly by the negative or repressive arm of the circadian clock) or of translation and transcription factors (mostly by the positive or activation arm of the circadian clock).^114^ Circadian rhythms and CVD are linked through multiple factors, with disruptions increasing the severity of cardiovascular risk factors and vice versa.^38,115^ Circadian rhythms can be disrupted at various levels, such as when clock input signals are misaligned with intrinsic molecular clocks, which can occur during jet lag or shift work.^116,117^ The present overview explores the connection between circadian rhythms and various factors affecting cardiovascular risk, including physiological modulators such as age, sex and pregnancy/lactation, comorbidities such as hypertension and metabolic disease, lifestyle factors such as smoking, alcohol intake and exercise, and environmental factors such as artificial light, temperature, air quality and noise (*Figure 3*).^38^ While an association is often observed in preclinical and clinical settings, a causal relationship with most factors remains to be established.

#### Physiological factors and comorbidities

Circadian misalignment in healthy adults leads to sex-specific changes in energy homeostasis, independent of behavioural or environmental factors,^118^ which are also observed following central circadian clock adaptations during pregnancy and lactation.^119^ Ageing affects the period and amplitude of circadian rhythms and melatonin levels, both of which decline later in life.^120^ Obesity and diabetes impact both circadian clock gene expression and function,^121^ and vice versa circadian rhythm disruption promotes metabolic disorders.^122^ CVD risk is also modulated by sex and age^123^ and cardiometabolic comorbidities,^124^ suggesting potential pathogenic contributions by circadian dysregulation. Inflammation and circadian rhythms are closely linked, with immune cell activity and pro-inflammatory cytokine levels showing 24 h fluctuations regulated by clock genes to maintain immune balance and tissue homeostasis. Chronic sleep deprivation and circadian disruption can amplify inflammation, increasing pro-inflammatory cytokines (IL-1β, IL-6, and tumour necrosis factor-α) and disrupting immune regulation, which are key contributors to CVD.^125–127^

#### Psychological and lifestyle factors

Depressive disorders are often associated with disrupted clock gene expression, resulting in depressive-prone features in rodent models.^128^ Mental stressors cause dysregulation of circadian rhythm through oxidative stress.^129^ Physical performance is influenced by circadian clock proteins, while inactivity or exercise can affect the circadian system.^130^ While exercise is a protective intervention in HF,^131^ benefits are also dependent on both time of the day and an individual's chronotype.^132^ Although exercise is generally regarded as highly protective, morningness persons may benefit more when exercising in the morning, whereas eveningness persons benefit from early or late exercise. Chronic alcohol consumption may disrupt molecular clocks and contribute to human alcohol-induced liver disease^133^ and addiction. Smoking alters gene expression of central and peripheral clock genes^134^ affecting sleep and circadian rhythms.^135^ Intermittent fasting improves health in part through benefits to circadian rhythm, and was found to decrease all-cause mortality and the risk of non-fatal MI.^136,137^ Nutrition and nutraceuticals can help reset peripheral circadian clocks.^138^ Shift work, jetlag and other sleep disorders disrupt the circadian clock system, which is associated with increased ischaemic heart damage upon MI.^139,140^ Thus, both well-accepted cardiovascular risk factors (depression and other mental disease,^141^ alcohol, smoking, unhealthy diet,^142^ and shiftwork) and protective factors (physical exercise, healthy diet and intermittent fasting) provide their effects at least in part via the circadian clock.

#### Environmental factors

Temperature oscillations, as small as 1°C, can alter the expression of circadian genes, affecting amplitude and phase.^143^ The circadian period length remains constant due to temperature compensation.^144^ Auditory function and sound stimuli, especially during the night, can also influence circadian rhythms through sleep deprivation, stress reactions (e.g. higher adrenaline and cortisol levels affect sleep) and oxidative stress (via redox modifications of clock core components and related phase/amplitude shifts).^145^ Light is a significant environmental signal that influences circadian rhythms, with detrimental effects of artificial light at night on sleep, stress hormones and oxidative stress,^146^ and seasonal changes affecting chronotype.^147^ Chemical pollutants, such as air pollution and heavy metals or pesticides can significantly alter circadian rhythms mostly by oxidative stress-dependent processes or direct interaction of the toxins with thiol-based enzymatic activity,^148,149^ affecting sleep-wake patterns and increasing CVD risk by causing oxidative stress.^150^ Extreme temperatures increase cardiovascular mortality^151^ and are a leading cause of death.^142^ Furthermore, transportation noise,^152^ air pollution,^142^ and toxic chemical in soil/water^152^ are leading risk factors for CVD or mortality.

### Chronomodulation of cardiovascular medicine

Chronotherapy considers the effect of circadian clock-driven rhythms in pharmacokinetic and pharmacodynamic processes, to minimalize adverse effects and optimize treatment efficacy.^153^ Moreover, it includes the circadian variation in disease pathophysiology influencing symptom intensity and time-at-risk as seen in MI, VA, and HF. Many cardiovascular drugs are prescribed at specific times as recommended by pharmaceutical companies, but the rationale is rarely disclosed. For example, short-acting statins are prescribed at bedtime to match cholesterol synthesis, whereas long-acting statins, with >24 h half-lives, do not necessitate circadian considerations. This principle extends to other drug classes, yet long-acting drugs may also be optimized through chronotherapy strategies depending on their pharmacodynamic profile.^154^

Hypertension management exemplifies the importance of circadian considerations in clinical practice. Disrupted BP rhythmicity, especially high morning surge and the absence of a nocturnal BP dip, are associated with increased risks for cardiovascular events, atrial stiffness and chronic kidney disease.^155–157^ Chronomodulated antihypertensive therapy aims at more precisely mitigating these risk factors, and has been studied in clinical trials. Initially the MAPEC study (2010)^158^ and HYGIA trial (2019)^159^ demonstrated significant reductions in major cardiac events (61% and 45%, respectively) with bedtime antihypertensive therapy. However, recent evidence provides a more nuanced perspective. A Cochrane systematic review and meta-analysis reported modest reductions in 24 h systolic (−1.34 mmHg, 95% confidence interval [CI]: −2.38 to −.30) and diastolic (−1.01 mmHg, 95% CI: −1.75 to −.27) BP and the morning surge following bedtime antihypertensives administration, but found no conclusive protective effect on cardiovascular outcomes.^160^ Similarly, no significant benefit or harm associated with bedtime use of one antihypertensive medicine was found in the BedMed trial, despite a substantial difference in nocturnal systolic BP.^161,162^ Overall, these studies suggest that administering antihypertensives upon waking or at bedtime is equally effective in primary care populations. However, important limitations include the short study duration (<6 months), lack of assessment of left ventricular hypertrophy, and absence of specific analysis for non-dipping hypertensive patients, despite previous evidence of benefit.^158^ Bedtime administration may still be considered for patients with specific risk factors comorbidities (e.g. IHD, non-dipping hypertension) or patient characteristics. For example, bedtime doses of long-acting diltiazem were associated with significantly greater reduction of mean systolic and diastolic BP in patients with moderate-to-severe essential hypertension,^9^ and an increased exercise tolerance in patients with angina pectoris^163^ compared to the morning dose. Moreover, accounting for endogenous circadian rhythms has shown potential for stratification and personalized tailoring of antihypertensive therapy to mitigate the risk of adverse cardiovascular events in a recent proof-of-concept study.^164^

Optimizing the timing of drug administration in cardiovascular therapy may offer benefits beyond BP control, potentially influencing cardiac remodelling. Circadian angiotensin expression suggests ACE inhibitor efficacy depends on dosing time. In a murine pressure-overload HF model, captopril administration during the rest period reduced cardiac remodelling and improved cardiac function without affecting BP.^165^

Diurnal coagulation and fibrinolysis fluctuations, resulting in hypercoagulability and hypofibrinolytic conditions during the morning hours, together with increased platelet activity, suggest an optimal anticoagulant timing.^166^ Evening rivaroxaban enhanced morning anticoagulation,^10^ while evening low-dose aspirin better suppressed the COX-1 platelet activity peak upon wakening.^166,167^ A clinical trial confirmed bedtime low-dose aspirin more effectively reduced morning platelet activity, particularly in women, correlating with improved outcomes in high-risk pregnancies.^168,169^

### Incorporation of circadian medicine in clinical care and research

Circadian medicine encompasses multiple facets, including the timing of treatment and diagnosis (exploiting), integrating patients’ circadian characteristics (detecting), and synchronizing or/and modifying the circadian system (targeting).^170^ However, a lack of awareness among medical professionals limits the integration of circadian strategies into routine patient care. Here, we provide a summary of opportunities and considerations for integration of circadian medicine in cardiovascular research.

Routinely monitored patient variables, such as core body temperature, HR, and BP, exhibit robust 24 h rhythms in healthy individuals.^171^ Interpreting these parameters with reference to the time of measurement is already common use. Additionally, since circadian rhythms in these variables are markers of health, assessing the degree of circadian disruption may offer valuable insights for diagnosing disease or determining prognosis. For example, evaluating the peak-to-trough excursions of physiological parameters in intensive care unit patients has shown that deviations of the circadian rhythm are associated with longer length of stay and higher in-hospital mortality.^172^ Similarly, a non-dipping BP phenotype revealed by 24 h measurements has been linked to worse disease outcomes, as discussed in section 3.^157^

The circadian variation of physiological processes is not limited to vital signs but extends to circulating factors in clinical laboratory measurements. For instance, cardiac biomarkers such as troponin-T,^173^ NT-proBNP^47^ and soluble ST2^174^ display circadian variation in plasma levels. Establishing time-dependent reference ranges for such biomarkers could help prevent both false-negative and false-positive results leading to under- and overdiagnosis, and in research settings would increase sensitivity for treatment effects.

To further assess the value of integrating circadian medicine into clinical routines, it is essential to determine the impact of timing on drug administration and interventional therapies (e.g. surgical procedures^29^) with respect to efficacy, safety, and healthcare costs. However, most studies conducted to date have significant limitations: (i) they are often small, single-centre studies, reducing reproducibility; (ii) they typically compare only two time points, whereas at least three are required to clearly demonstrate peak/trough variation; and (iii) they fail to account for individual circadian phase as a potential confounder (e.g. seasonal variability, working hours/shift work, transmeridian travel, and chronotype including food intake and exercise). Investigating individual variability and identification of healthy people at risk is increasingly feasible with the widespread use of wearable devices, which provide extensive data on total activity, timing of physical activity, posture, HR (and variability), sleep quality, and body temperature. Additionally, recent advancements in digital circadian assessment tools, such as TimeTeller,^175,176^ enable accurate determination of individual circadian rhythms and their strength. Integrating these circadian metrics, with the established chronotype assessment tools (e.g. questionnaire), into clinical care represents a significant opportunity to enhance patient management and optimize the efficiency of clinical studies.

## Conclusions

In conclusion, circadian rhythms play a crucial role in cardiovascular health, influencing autonomic balance, cardiac function, and CVD progression. Disruptions increase the risk of IHD, HF, and arrhythmias, highlighting their role in CVD pathophysiology. By aligning interventions with the natural circadian cycle, it may be possible to enhance the efficacy of cardiovascular treatments, reduce side effects, and potentially improve long-term outcomes. Multiple factors related to the circadian clock can be considered to enhance quality or efficiency of clinical care, future clinical studies, and drug development.

## Supplementary Material

ehaf367_Supplementary_Data

## Declarations

### Data Availability

No data were generated or analysed for or in support of this paper.
