# Supplementary material for: Circadian rhythms in cardiovascular disease
Source: Eur Heart J. 2025 Jul 15;46(36):3532–45. doi: 10.1093/eurheartj/ehaf367 (PMC12450527; doi:10.1093/eurheartj/ehaf367)
Supplement: ehaf367_Supplementary_Data [file ehaf367_supplementary_data.docx]

**Supplemental material**

Circadian rhythms in cardiac disease: state-of-the-art review

**Content**

**Supplemental methods**

Search strategy, selection criteria and selection process– Page 2

**Supplemental results**

Supplemental Table 1: Search terms for the search strategy, including MeSH and synonyms – Page 3

Supplemental Table 2: Studies analysing the daily variation in heart failure, myocardial infarction and infarct size – Page 4-5

Supplemental Table 3: Studies analysing the daily variation in ventricular arrhythmia onset and sudden cardiac death. – Page 6-7

Supplemental Table 4: Studies analysing the daily variation in atrial fibrillation onset. – Page 8-9

**Supplemental references -** Page 10-14

**Supplemental methods**

This narrative review was performed with a systematic approach regarding search strategy and study selection for the sections on cardiac disease.

Search strategy and selection criteria

We used PubMed to search for literature in the Medical Analysis and Retrieval System Online (MEDLINE) database on October 17th, 2024. The search string was built using Medical Subject Headings (MeSH) terms as well as synonyms for title abstract screening. Each search string consisted of 1) type of cardiac disease (i.e., ischemic heart disease, arrhythmias, heart failure and chemotherapy-induced cardiac disease), along with 2) circadian rhythm or chronomodulation (Supplemental table 1). These were used to search for studies published in the English language and without a restriction on publication date.

Studies evaluating the incidence, diagnosis, aetiology, prognosis and therapy of cardiac disease and its relation to circadian rhythm were selected upon meeting the following criteria: (1) trials, population-based cohort or registry studies on the specific type of cardiac disease; (2) circadian rhythm was the determinant of interest, and; (3) studies included adults, aged ≥ 18 years. This review focused on the clinical aspects of the field and therefore excluded pre-clinical studies.

Selection Process

For the study selection process, we used Rayyan, a web-based AI powered tool for systematic literature reviews (Rayyan Systems Inc., Cambridge, USA). Two researchers (IRK, YK) performed the title and abstract screening, independently of each other. Where needed articles were discussed until consensus was reached. Afterwards, full-text screening was performed by one researchers (IRK or YK), and the extracted information was used for the main text of the review. The initial search identified a total of 5.019 articles from MEDLINE using PubMed for abstract assessment. In Rayyan a total of 76 duplicates were removed. After screening using the inclusion criteria a remaining 481 articles were assessed for this review. Furthermore, additional studies were selected with the snowballing technique, which involves identifying relevant studies from the reference lists of previously included articles. In snowballing, the reference lists of key papers are evaluated to select additional studies that are related to the research topic, ensuring that the review is as comprehensive as possible by including studies that may not have been captured in the primary search.

**Supplemental Table 1: Search terms for the search strategy, including MeSH and synonyms**

| **Search terms** | **Synonyms MeSH and Title-Abstract** |
| --- | --- |
| Circadian rhythm | Circadian clock, chronobiology, biological clock, circadian cycle, circadian rhythm, 24-hour rhythm, circadian variation, circadian |
| Chronomodulation | Chronotherapies, drug, chronotherapy, circadian therapy, timing-specific therapy, time-based treatment, circadian modulation, chronobiological intervention, chronotherapeutic strategy, chronomodulation |
| Ischemic heart disease | Ischemic heart disease, coronary heart disease, myocardial ischemia, ischemic heart condition, atherosclerotic heart disease, atherosclerosis, atherosclerosis, myocardial infarction |
| Arrhythmias | Arrhythmia, cardiac arrhythmia, atrial fibrillation, ventricular fibrillation, bradycardia, tachycardia, heart rhythm disorder, cardiac dysrhythmias, long QT syndrome, sick sinus syndrome, atrial flutter, PVCs, supraventricular tachycardia, premature ventricular contractions, atrial ectopic tachycardia, sudden cardiac death |
| Heart failure | Heart failure, congestive heart failure, diastolic heart failure, systolic heart failure, heart failure with preserved ejection fraction, heart failure with reduced ejection fraction, acute heart failure, chronic heart failure, decompensatio cordis, cardiac decompensation |
| Chemotherapy-induced cardiac disease | chemotherapy-related cardiotoxicity, cardiotoxic agents, chemotherapy-induced cardiomyopathy, cancer therapy-related cardiac dysfunction, cardiotoxicity, cardiotoxic effects of chemotherapy, cancer therapy-related cardiac dysfunction, chemotherapy-associated heart disease, cardiovascular disease, cardiac disease, heart disease, cardiac, cancer, cancer therapy, chemotherapy, chemotherapeutic agents. |
| The terminology included both singular and plural forms, as well as word combinations (e.g., "therapy" and "treatment" were used interchangeably) | |

**Supplemental Table 2. Studies analysing the daily variation in heart failure, myocardial infarction and infarct size.**

| **Author** | **Year** | **N** | **Population** | **Endpoint** | **High risk time** | **Low risk time** |
| --- | --- | --- | --- | --- | --- | --- |
| *Heart failure* | | | | | | |
| Allegra^48^ | 2001 | 2 370 233 | Not specified | Emergency department visit for congestive heart failure | 08.00-15.00 | 19.00-8.00 |
| Seneviratna^49^ | 2015 | 6 710 | Male 79%, mean age 60yr | Acute heart failure (after STEMI) | 00.00-06.00 * | 06.00-12.00 * |
| Paradies^50^ | 2020 | 11 731 | Male 85%, mean age 58yr | Heart failure hospitalization (at 1 year, after STEMI) | 00.00-06.00 * |  |
| *Myocardial infarction* | | | | | | |
| Willich^21^ | 1989 | 1 741 | Male 82%, ≥60yr 48% | Incidence MI | 06.00-12.00, | 00.00-01.00 |
|  |  |  |  |  | Peak 08.00-09.00 |  |
| Suarez-Barrientos^51^ | 2011 | 811 | Male 78%; mean age 62yr | Incidence STEMI | 06.00-12.00 |  |
| Mogabgab^52^ | 2012 | 35 492 | Male 76%, mean age 61yr; | Incidence STEMI | 06.00-14.00 | 22.00-06.00 |
| Fournier^53^ | 2012 | 353 | Male 73%, mean age 65yr | Incidence MI | 08.00-15.00 |  |
| Mohammad^54^ | 2018 | 283 014 | Male 64%; mean age 72yr | Incidence MI (predominantly nSTEMI) | Peak 08.00 |  |
| Nordenskjold^55^ | 2019 | 9 092 | Male 38%; mean age 66yr | Incidence of MINOCA | 06.00-18.00, | 00.00-06.00 |
|  |  |  |  |  | Peak 08.00 |  |
| Mahajan^56^ | 2019 | 322 523 | Male 65%; ≥60yr 56% | Incidence of MINOCA and obstr. MI | 08.00-14.00 |  |
| Peng | 2020 | 1 099 | Male 78%; mean age 62yr | Incidence STEMI | 06.00-12.00 |  |
| Araki^57^ | 2021 | 648 | Male 80%, mean age 65yr | Incidence STEMI | 07.00-10.00 |  |
|  |  |  |  | Plaque rupture ** | 06.00-18.00 |  |
|  |  |  |  |  | Peak 09.00 *** |  |
| Daios^43^ | 2021 | 178 | DM, Male 69%, mean age 68yr | Incidence MI | 02.00-03.00, |  |
|  |  |  |  |  | 14.00-15.00, |  |
|  |  |  |  |  | 22.00-23.00 |  |
| Xin^58^ | 2022 | 3 158 | Male 73%, mean age 61yr | Incidence MI | 06.00-12.00 |  |
| Chan^59^ | 2023 | 865 | Male 84%, mean age 60yr | Incidence MI | 06.00-12.00 | 00.00-06.00 |
| *Infarct size* | | | | | | |
| Suarez-Barrientos^51^ | 2011 | 811 | Male 78%, mean age 62yr | Myocardial infarct size, defined as peak CK and TnI levels | 06.00-12.00 |  |
| Reiter^60^ | 2012 | 165 | Male 79%, mean age 60yr | Peak CK and infarct size by CMR based on AAR | Peak 01.00 |  |
| Fournier^53^ | 2012 | 353 | Male 73%, mean age 65yr | Highest myocardial injury (peak CK levels) for onset time | 00.00-06.00 |  |
| Seneviratna | 2015 | 6 710 | Male 79%, mean age 60yr | Highest myocardial injury (peak CK levels) for onset time | 00.00-06.00 | 06.00-12.00 |
| Ari^61^ | 2016 | 252 | Male 83%; mean age 57yr | Peak CK and peak systolic myocardial velocities (TDI echocardiography) | 06.00-12.00 |  |
| Bulluck^62^ | 2017 | 169 | Male 51%; mean ag 59yr | Myocardial infarct size by CMR, expressed as % of the AAR | 00.00-01.00 | 12.00-13.00 |
| STEMI: ST-Segment Elevation Myocardial Infarction, nSTEMI: Non-ST-Segment Elevation Myocardial Infarction, MI: Myocardial Infarction, DM: Diabetes Mellitus, CK: Creatine Kinase, TnI: Troponin I, TDI: Tissue Doppler Imaging, CMR: Cardiac Magnetic Resonance, AAR: Area at Risk, * STEMI onset time, ** No daily variation in STEMI due to plaque erosion and calcified plaque, *** Only on weekdays | | | | | | |

**Supplemental Table 3.** **Studies analysing the daily variation in ventricular arrhythmia onset and sudden cardiac death.**

| **Author** | **Year** | **N** | | **Variables quantified** | | | **Population** | | **Type of arrhythmia** | | **Time of peak** | | **Time of second peak** | |  |
| --- | --- | --- | --- | --- | --- | --- | --- | --- | --- | --- | --- | --- | --- | --- | --- |
| *Ventricular Arrhythmias* | | | | | | | | | | | | | | |  |
| Twidale^1^ | 1989 | 68 | | Holter | | | Male 93%; IHD 80% | | VTA | | 06:00-12:00 | |  | |  |
| Lanza^2^ | 1990 | 38 | | Holter | | | Male 80%; Healthy 50%, IHD 50% | | VPC | | 11:00-13:00 | | Late afternoon | |  |
| Lampert^3^ | 1994 | 32 | | ICD | | | Male 75%; IHD 100% BB 37% | | VTA | | 06:00-12:00 | |  | |  |
| Tofler^4^ | 1995 | 483 | | ICD | | | Male 84%; IHD (80%) | | VTA | | 09:00-12:00 | |  | |  |
| Wood^5^ | 1995 | 43 | | ICD | | | Male 86%; IHD (80%); BB 7% | | VTA | | 14:00-17:00 | |  | |  |
| d’Avila^6^ | 1995 | 22 | | ICD | | | Male 82%; IHD 75% BB 0% | | ICD Shocks | | 10:00-11:00 | |  | |  |
| Mallavarapu^7^ | 1995 | 390 | | ICD | | | Male 90%; IHD | | VA | | 10:00-12:00 | |  | |  |
| Behrens^8^ | 1995 | 39 | | ICD | | | Male 95%; IHD 80% | | ICD Shocks | | 08:00-11:00 | | 16:00-20:00 | |  |
| Behrens^9^ | 1997 | 87 | | ICD | | | Male 94%; 70% IHD; BB 20% | | ICD Shocks | | 06:00-11:00 | |  | |  |
| Englund^10^ | 1999 | 310 | | ICD | | | Male 85%; CD 66%; BB 10% | | VA | | 07:00-10:00 | | 16:00-18:00 | |  |
| Peters^11^ | 1999 | 683 | | ICD | | | Male 82%; IHD 75%; iDCM 15%; 33% anti-arrhythmic drugs; | | VA | | 09:00-18:00 | |  | |  |
| Kozak^12^ | 2003 | 72 | | ICD | | | Male 70%; IHD 80% | | VA | | 07:00-11:00 | | 18:00-19:00 | |  |
| Anand^13^ | 2007 | 154 | | ICD | | | Male 78%; IHD 71% | | VA | | 08:00-13:00 | | 17:00-22:00 | |  |
| Eksik^14^ | 2007 | 70 | | ICD | | | Male 90%; IHD 76%; BB 7% | | VA | | 06:00-12:00 | |  | |  |
| Maron^15^ | 2009 | 63 | | ICD | | | Male 71%; HCM | | VA | | 12:00-00:00 | |  | |  |
| Patton^16^ | 2014 | 811 | | ICD | | | Male 77%; IHD 50% | | VA | | No morning peak* | | | |  |
| Ruwald^17^ | 2015 | 1790 | | ICD | | | Male 75%; IHD 60%; BB 90% | | VTA | | 07:00-11:00 | | 18:00-22:00 | |  |
| Maan^18^ | 2019 | 3969 | | ICD | | | Male 77%; IHD 67% | | VA | | 08.00-22.00 | |  | |  |
| Li^19^ | 2020 | 1559 | | ICD | | | Military veterans Male 99%; IHD 70%; BB 90% | | VTA | | 14:00-17:00 | | 07:0-10:00 | |  |
| Vergara^20^ | 2021 | 446 | | ICD | | | Male 84%; IHD 44% | | VA | | 08.00-16.00 | |  | |  |
| *Sudden Cardiac Death* | | |  | |  |  | |  | |  | |  | |  | |
| Willich^21^ | 1987 | 5209 | | EMS data | | | General population | |  | | 07:00-09:00 | |  | |  |
| Muller^22^ | 1987 | 2203 | | ICD codes | | | General population | |  | | 07:00-11:00 | | 17:00-18:00 | |  |
| Willich^23^ | 1992 | 94 | | EMS data & Interviews | | | General population | |  | | 09:00-12:00 | |  | |  |
| Levine^24^ | 1992 | 1019 | | EMS data | | | General population | |  | | 06:00-12:00 | |  | |  |
| Moser^25^ | 1994 | 566 | | Registry Data | | | HF pts (IHD & N-IHD) | |  | | 06:00-12:00 | |  | |  |
| Arntz^26^ | 2000 | 24061 | | SCD | | | General population | |  | | 06:00-12:00 | |  | |  |
| Nakanishi^27^ | 2011 | 2396 | | OHCA | | | General population | |  | | 8:00 and 19:00 | |  | |  |
| Lopez^28^ | 2012 | 1286 | | Registry Data | | | General population | |  | | 10:00-13:00 | | 18:00-21:00 | |  |
| Ni^29^ | 2019 | 1535 | | Witnessed SCA | | | General population | |  | | No morning peak* | | | |  |
| BB - Current therapy including beta-blockers; ICD - Implantable cardioverter-defibrillator; iDCM – Idiopathic Dilated Cardiomyopathy; IHD - Ischemic heart disease; N - Number of subjects; USA - United States of America; VA - Ventricular tachyarrhythmia; VPC - Ventricular premature contraction; VTA - Ventricular tachyarrhythmia; % of population with described characteristic; * Only usual nadir in early morning. | | | | | | | | | | | | | | |  |

**Supplemental Table 3. Studies analysing the daily variation in paroxysmal and persistent/sustained atrial fibrillation onset.**

| **Author** | **Year** | **N** | **Mode of detection** | **Population** | **Time of peak** | **Time of second peak** |
| --- | --- | --- | --- | --- | --- | --- |
| Paroxysmal | | | | | | |
| Clair^30^ | 1993 | 37 | Onset of symptoms | Mean age 40yr; 30% SHD | No clear peak |  |
| Rostagno^31^ | 1993 | 776 | Onset of symptoms | Symptomatic pts cardiac care unit | 00:00-04:00 | 08:00-12:00 |
| Viskin^32^ | 1999 | 3343 | Onset of symptoms | Mean age 74yr; Emergency calls; Majority SHD; New onset AF | 07:00-10:00 | 18:00-21:00 |
| Gillis^33^ | 2001 | 67 | Implantable device | Mean age 60yr; Pts scheduled for AV nodal ablation (refractory to AD); | 04:00-07:00 | 16:00-19:00 |
| Delle Karth^34^ | 2003 | 55 | Continues monitoring | Mean age: 65yr; ICU patients; New-onset AF | 08:00-16:00 |  |
| Mitchell^35^ | 2003 | 15 | Holter | Mean age 63yr; 50% SHD | 11:00-22:00 |  |
| Vincenti^36^ | 2006 | 90 | Holter | Mean age 67yr; 31% SHD | 12:00-14:00 and 18:00-02:00 | 04:00-06:00 |
| Deguchi^37^ | 2009 | 217 | Holter | Mean age 65yr; SHD | 05:00-08:00 | 18:00 and 00:00 |
| Shusterman^38^ | 2012 | 236 | Implantable device | Mean age 63yr; 33% IHD; 90% BB; 100% AD | 23:00-04:00 |  |
| Capucci^39^ | 2012 | 119 | Implantable device | Mean age 78yr; Sick sinus syndrome patients | 04:00-05:00 | 15:00-19:00 |
| Kim^40^ | 2020 | 74 | Holter | Mean age 80yr; 10% SHD; 66% AD | 15:00-18:00 |  |
| Younis^41^ | 2020 | 66 | Implantable device | Mean age 60yr; HF patients; New-onset AF | 17:00-23:00 | 12:00-17:00 |
| Bedford^42^* | 2021 | 2017 | Continues monitoring | Mean age 72; ICU patients; New onset AF | 08:00 and 20:00 | |
| Daios^43^* | 2021 | 173 | Onset of symptoms | Mean age 67yr; DM pts cardiac care unit; new onset | 12:00-14:00 |  |
| Lin^44^ | 2022 | 66 | Onset of symptoms | Mean age 60yrs; ED; non-OSA | 22:00-00:00; | 08:00-10:00 |
|  |  |  |  | Mean age 60yrs; ED; OSA | 04:00-10:00 |  |
| Tas^45^ | 2022 | 23 | Holter | Mean age 60yr; no SHD or AD | 08:00-09:00 | 16:00-17:00 |
| van de Lande^46^ | 2024 | 278 | Implantable device | Mean age 66 yr; no SHD; 50% BB | No clear peak; 30% had a clear pattern (nocturnal or daytime); | |
| *Persistent/sustained SVT* | | | | | | |
| Kupari^47^ | 1990 | 152 | Onset of symptoms | Age <65yr; ED; 25% BB | 18:00-00:00 | 06:00-12:00 |
| Mitchell^35^ | 2003 | 15 | Holter | Mean age 63yrs; 50% SHD | 20:00-08:00 |  |
| Implantable devices include: ILR, CRTD, and ICDs. AD – Anti-arrhythmic drugs; AF – Atrial fibrillation; AV – Atrioventricular; BB – Beta-blocker; CRTD – Cardiac resynchronization therapy Defibrillator; DM – Diabetes Mellitus; ED – Emergency department; HF – Heart Failure; ICD – Implantable Cardioverter-Defibrillator; ICU – Intensive Care Unit; ILR – Implantable Loop Recorder; OSA – Obstructive Sleep Apnea; SHD – Structural Heart Disease. *Retrospective studies | | | | | | |

**Supplemental references**

1. Twidale N, Taylor S, Heddle WF, Ayres BF, Tonkin AM. Morning increase in the time of onset of sustained ventricular tachycardia. *The American Journal of Cardiology*. 64(18);1989:1204-1206.

2. Lanza GA, Cortellessa MC, Rebuzzi AG, et al. Reproducibility in circadian rhythm of ventricular premature complexes. *The American Journal of Cardiology*. 66(15);1990:1099-1106.

3. Lampert R, Rosenfeld L, Batsford W, Lee F, McPherson C. Circadian variation of sustained ventricular tachycardia in patients with coronary artery disease and implantable cardioverter-defibrillators. *Circulation*. 90(1);1994:241-247.

4. Tofler GH, Gebara OCE, Mittleman MA, et al. Morning Peak in Ventricular Tachyarrhythmias Detected by Time of Implantable Cardioverter/Defibrillator Therapy. *Circulation*. 92(5);1995:1203-1208.

5. Wood MA, Simpson PM, London WB, et al. Circadian pattern of ventricular tachyarrhythmias in patients with implantable cardioverter-defibrillators. *Journal of the American College of Cardiology*. 25(4);1995:901-907.

6. D’AVILA A, WELLENS F, ANDRIES E, BRUGADA P. At what time are implantable defibrillator shocks delivered?: Evidence for individual circadian variance in sudden cardiac death. *European Heart Journal*. 16(9);1995:1231-1233.

7. Mallavarapu C, Pancholy S, Schwartzman D, et al. Circadian variation of ventricular arrhythmia recurrences after cardioverter-defibrillator implantation in patients with healed myocardial infarcts. *The American Journal of Cardiology*. 75(16);1995:1140-1144.

8. Behrens S, Galecka M, Brüggemann T, et al. Circadian variation of sustained ventricular tachyarrhythmias terminated by appropriate shocks in patients with an implantable cardioverter defibrillator. *American Heart Journal*. 130(1);1995:79-84.

9. Behrens S, Ehlers C, Brüggemann T, et al. Modification of the circadian pattern of ventricular tachyarrhythmias by beta-blocker therapy. *Clinical Cardiology*. 20(3);1997:253-257.

10. Englund A, Behrens S, Wegscheider K, Rowland E. Circadian variation of malignant ventricular arrhythmias in patients with ischemic and nonischemic heart disease after cardioverter defibrillator implantation. *Journal of the American College of Cardiology*. 34(5);1999:1560-1568.

11. Peters RW, McQuillan S, Gold MR. Interaction of septadian and circadian rhythms in life-threatening ventricular arrhythmias in patients with implantable cardioverter-defibrillators. *The American Journal of Cardiology*. 84(5);1999:555-557.

12. Kozák M, Křivan L, Semrád B. Circadian Variations in the Occurrence of Ventricular Tachyarrhythmias in Patients with Implantable Cardioverter Defibrillators. *Pacing and Clinical Electrophysiology*. 26(3);2003:731-735.

13. Anand K, Aryana A, Cloutier D, et al. Circadian, Daily, and Seasonal Distributions of Ventricular Tachyarrhythmias in Patients With Implantable Cardioverter-Defibrillators. *The American Journal of Cardiology*. 100(7);2007:1134-1138.

14. Eksik A, Akyol A, Norgaz T, et al. Circadian pattern of spontaneous ventricular tachyarrhythmias in patients with implantable cardioverter defibrillators. *Medical Science Monitor*. 13(9);2007:CR412-CR416.

15. Maron BJ, Semsarian C, Shen WK, et al. Circadian patterns in the occurrence of malignant ventricular tachyarrhythmias triggering defibrillator interventions in patients with hypertrophic cardiomyopathy. *Heart Rhythm*. 6(5);2009:599-602.

16. Patton KK, Hellkamp AS, Lee KL, et al. Unexpected deviation in circadian variation of ventricular arrhythmias: the SCD-HeFT (Sudden Cardiac Death in Heart Failure Trial). *Journal of the American College of Cardiology*. 63(24);2014:2702-2708.

17. Ruwald MH, Moss AJ, Zareba W, et al. Circadian Distribution of Ventricular Tachyarrhythmias and Association with Mortality in the MADIT-CRT Trial. *Journal of Cardiovascular Electrophysiology*. 26(3);2015:291-299.

18. Maan A, Sherfesee L, Lexcen D, Heist EK, Cheng A. Diurnal, Seasonal, and Monthly Variations in Ventricular Arrhythmias in Patients With Implantable Cardioverter-Defibrillators. *JACC: Clinical Electrophysiology*. 5(8);2019:979-986.

19. Li Y, Nantsupawat T, Tholakanahalli V, et al. Characteristics and periodicity of sustained ventricular tachyarrhythmia events in a population of military veterans with implantable cardioverter defibrillator. *Journal of Interventional Cardiac Electrophysiology*. 58(2);2020:123-132.

20. Vergara P, Pignalberi C, Pisanò EC, et al. Circadian periodicity affects the type of ventricular arrhythmias and efficacy of implantable defibrillator therapies. *Journal of Cardiovascular Electrophysiology*. 32(9);2021:2528-2535.

21. Willich SN, Levy D, Rocco MB, Tofler GH, Stone PH, Muller JE. Circadian variation in the incidence of sudden cardiac death in the framingham heart study population. *The American Journal of Cardiology*. 60(10);1987:801-806.

22. Muller JE, Ludmer PL, Willich SN, et al. Circadian variation in the frequency of sudden cardiac death. *Circulation*. 75(1);1987:131-138.

23. Willich SN, Goldberg RJ, Maclure M, Perriello L, Muller JE. Increased onset of sudden cardiac death in the first three hours after awakening. *The American Journal of Cardiology*. 70(1);1992:65-68.

24. Levine RL, Pepe PE, Fromm RE Jr, et al. Prospective Evidence of a Circadian Rhythm for Out-of-Hospital Cardiac Arrests. *JAMA*. 267(21);1992:2935-2937.

25. Moser DK, Stevenson WG, Woo MA, Stevenson LW. Timing of sudden death in patients with heart failure. *Journal of the American College of Cardiology*. 24(4);1994:963-967.

26. Arntz HR, Willich SN, Schreiber C, Brüggemann T, Stern R, Schultheiss HP. Diurnal, weekly and seasonal variation of sudden death. Population-based analysis of 24,061 consecutive cases. *European Heart Journal*. 21(4);2000:315-320.

27. Nakanishi N, Nishizawa S, Kitamura Y, et al. Circadian, weekly, and seasonal mortality variations in out-of-hospital cardiac arrest in Japan: analysis from AMI-Kyoto Multicenter Risk Study database. *The American Journal of Emergency Medicine*. 29(9);2011:1037-1043.

28. López-Messa JB, Alonso-Fernández JI, Andrés-de Llano JM, et al. Circadian rhythm and time variations in out-hospital sudden cardiac arrest. *Medicina Intensiva (English Edition)*. 36(6);2012:402-409.

29. Ni YM, Rusinaru C, Reinier K, et al. Unexpected Shift in Circadian and Septadian Variation of Sudden Cardiac Arrest: The Oregon Sudden Unexpected Death Study. *Heart rhythm*. 16(3);2019:411-415.

30. Clair WK, Wilkinson WE, McCarthy EA, Page RL, Pritchett EL. Spontaneous occurrence of symptomatic paroxysmal atrial fibrillation and paroxysmal supraventricular tachycardia in untreated patients. *Circulation*. 87(4);1993:1114-1122.

31. Rostagno C, Taddei T, Paladini B, Modesti PA, Utari P, Bertini G. The onset of symptomatic atrial fibrillation and paroxysmal supraventricular tachycardia is characterized by different circadian rhythms. *The American Journal of Cardiology*. 71(5);1993:453-455.

32. Viskin S, Golovner M, Malov N, et al. Circadian variation of symptomatic paroxysmal atrial fibrillation. Data from almost 10 000 episodes. *European Heart Journal*. 20(19);1999:1429-1434.

33. Gillis AM, Connolly SJ, Dubuc M, et al. Circadian variation of paroxysmal atrial fibrillation. *American Journal of Cardiology*. 87(6);2001:794-798.

34. Delle Karth G, Reinelt P, Buberl A, et al. Circadian variation in ventricular tachycardia and atrial fibrillation in a medical-cardiological ICU. *Intensive Care Medicine*. 29(6);2003:963-968.

35. Mitchell ARJ, Spurrell PAR, Sulke N. Circadian variation of arrhythmia onset patterns in patients with persistent atrial fibrillation. *American Heart Journal*. 146(5);2003:902-907.

36. Vincenti A, Brambilla R, Fumagalli MG, Merola R, Pedretti S. Onset mechanism of paroxysmal atrial fibrillation detected by ambulatory Holter monitoring. *EP Europace*. 8(3);2006:204-210.

37. Deguchi Y, Amino M, Adachi K, et al. Circadian distribution of paroxysmal atrial fibrillation in patients with and without structural heart disease in untreated state. *Annals of Noninvasive Electrocardiology: The Official Journal of the International Society for Holter and Noninvasive Electrocardiology, Inc*. 14(3);2009:280-289.

38. Shusterman V, Warman E, London B, Schwartzman D. Nocturnal peak in atrial tachyarrhythmia occurrence as a function of arrhythmia burden. *Journal of Cardiovascular Electrophysiology*. 23(6);2012:604-611.

39. Capucci A, Calcagnini G, Mattei E, et al. Daily distribution of atrial arrhythmic episodes in sick sinus syndrome patients: implications for atrial arrhythmia monitoring. *Europace: European Pacing, Arrhythmias, and Cardiac Electrophysiology: Journal of the Working Groups on Cardiac Pacing, Arrhythmias, and Cardiac Cellular Electrophysiology of the European Society of Cardiology*. 14(8);2012:1117-1124.

40. Kim J, Wang W, Norby FL, et al. Diurnal circadian variations in paroxysmal atrial fibrillation: The atherosclerosis risk in communities (ARIC) study. *Journal of Electrocardiology*. 63;2020:98-103.

41. Younis A, Goldenberg I, McNitt S, et al. Circadian variation and seasonal distribution of implantable defibrillator detected new onset atrial fibrillation. *Pacing and clinical electrophysiology: PACE*. 43(12);2020:1495-1500.

42. Bedford JP, Redfern O, Johnson A, Rajappan K, Watkinson PJ. Circadian variation in new-onset atrial fibrillation in patients in ICUs. *Journal of Critical Care*. 67;2022:1-2.

43. Daios S, Savopoulos C, Kanellos I, et al. Circadian Pattern of Acute Myocardial Infarction and Atrial Fibrillation in a Mediterranean Country: A study in Diabetic Patients. *Medicina (Kaunas, Lithuania)*. 57(1);2021:41.

44. Lin CH, Timofeeva M, O ’Brien Tara, Lyons OD. Obstructive sleep apnea and nocturnal attacks of paroxysmal atrial fibrillation. *Journal of Clinical Sleep Medicine*. 18(5);2022:1279-1286.

45. Taş Ü, Taş S, Yavuz İ. The relationship between morning blood pressure surge and asymptomatic episodes of paroxysmal atrial fibrillation in patients with systemic arterial hypertension. *Turkish Journal of Medical Sciences*. 52(6);2022:1906-1916.

46. van de Lande ME, Rama RS, Koldenhof T, et al. Time of onset of atrial fibrillation and atrial fibrillation progression data from the RACE V study. *Europace: European Pacing, Arrhythmias, and Cardiac Electrophysiology: Journal of the Working Groups on Cardiac Pacing, Arrhythmias, and Cardiac Cellular Electrophysiology of the European Society of Cardiology*. 25(5);2023:euad058.

47. Kupari M, Koskinen P, Leinonen H. Double-peaking circadian variation in the occurrence of sustained supraventricular tachyarrhythmias. *American Heart Journal*. 120(6, Part 1);1990:1364-1369.

48. Allegra JR, Cochrane DG, Biglow R. Monthly, Weekly, and Daily Patterns in the Incidence of Congestive Heart Failure. *Academic Emergency Medicine*. 8(6);2001:682-685.

49. Seneviratna A, Lim GH, Devi A, et al. Circadian Dependence of Infarct Size and Acute Heart Failure in ST Elevation Myocardial Infarction. *PLOS ONE*. 10(6);2015:e0128526.

50. Paradies V, Zheng H, Chan MHH, et al. Impact of time of onset of symptom of ST-segment elevation myocardial infarction on 1-year rehospitalization for heart failure and mortality. *American Heart Journal*. 224;2020:1-9.

51. Suárez-Barrientos A, López-Romero P, Vivas D, et al. Circadian variations of infarct size in acute myocardial infarction. *Heart*. 97(12);2011:970-976.

52. Mogabgab O, Giugliano RP, Sabatine MS, et al. Circadian Variation in Patient Characteristics and Outcomes in ST-Segment Elevation Myocardial Infarction. *Chronobiology International*. 29(10);2012:1390-1396.

53. Fournier S, Eeckhout E, Mangiacapra F, et al. Circadian variations of ischemic burden among patients with myocardial infarction undergoing primary percutaneous coronary intervention. *American Heart Journal*. 163(2);2012:208-213.

54. Mohammad MA, Karlsson S, Haddad J, et al. Christmas, national holidays, sport events, and time factors as triggers of acute myocardial infarction: SWEDEHEART observational study 1998-2013. *BMJ*. 363;2018:k4811.

55. Nordenskjöld AM, Eggers KM, Jernberg T, Mohammad MA, Erlinge D, Lindahl B. Circadian onset and prognosis of myocardial infarction with non-obstructive coronary arteries (MINOCA). *PLOS ONE*. 14(4);2019:e0216073.

56. Mahajan AM, Gandhi H, Smilowitz NR, et al. Seasonal and circadian patterns of myocardial infarction by coronary artery disease status and sex in the ACTION Registry-GWTG. *International Journal of Cardiology*. 274;2019:16-20.

57. Araki M, Yonetsu T, Kurihara O, et al. Circadian variations in pathogenesis of ST-segment elevation myocardial infarction: an optical coherence tomography study. *Journal of Thrombosis and Thrombolysis*. 51(2);2021:379-387.

58. Xin M, Zhang S, Zhao L, Jin X, Kim W, Cheng XW. Circadian and seasonal variation in onset of acute myocardial infarction. *Medicine*. 101(28);2022:e29839.

59. Chan B, Buckley T, Hansen P, Shaw E, Tofler GH. Circadian variation in acute myocardial infarction and modification by coronary artery disease: a prospective observational study. *European Heart Journal Open*. 3(4);2023:oead068.

60. Reiter R, Swingen C, Moore L, Henry TD, Traverse JH. Circadian Dependence of Infarct Size and Left Ventricular Function After ST Elevation Myocardial Infarction. *Circulation Research*. 110(1);2012:105-110.

61. Arı H, Sonmez O, Koc F, et al. Circadian Rhythm of Infarct Size and Left Ventricular Function Evaluated with Tissue Doppler Echocardiography in ST Elevation Myocardial Infarction. *Heart, Lung and Circulation*. 25(3);2016:250-256.

62. Bulluck H, Nicholas J, Crimi G, et al. Circadian variation in acute myocardial infarct size assessed by cardiovascular magnetic resonance in reperfused STEMI patients. *International Journal of Cardiology*. 230;2017:149-154.
